# House and Home

**Published:** 1893

**Authors:** Lillian White


### HOUSE AND HOME.

Conducted by Lillian White.

### THE HOUSE.

A Wheat Sachet.—Did you know a bunch of wheat may be made into a very pretty sachet? Take a small bunch, say about twelve stalks, and tie the heads together. Then make a small, oval bag of pink China silk. Fill this with cotton sprinkled with sachet powder. Arrange the stalks of wheat about the bag and run light blue and mauve baby ribbon in and out over them until they form a firm enclosure for it. Fasten a blue ribbon at one end and one of mauve at the other. Tie them in a bow and hang the sachet up. Such sachets may be bought for 60 cents, but made at home they need not cost over a quarter.

A Darning Hint.—Unless a woman has the peculiar temperament which would cut holes in stockings for the sake of making a "beautiful darn," she will do well to adopt this precaution with all her hose. Before putting them on at all, "run" them with soft darning cotton throughout the entire length of heel, toe and sole. They should not be closely run nor should the thread be tightly drawn. This simple process will keep the stockings from breaking far beyond the usual period.

Corduroy Coverings.—Those hygienic housewives who dislike upholstered furniture and have a fondness for rattan and portable cushions are turning their attention to corduroy as a winter covering for cushions. Its claims to popular favor are many. In the first place, it is fairly inexpensive. Then it has the wearing qualities of sheet-iron. It comes in all colors. A drawing-room furnished in wicker and corduroy of dark green and brown shades is charming. The chair seats and backs are made by an upholsterer and are tufted and corded properly. The long wicker lounge has a three-part cover of brown corduroy and the usual embankment of bright-colored silk pillows.

In White and Silver.—She was not a rich woman, but as she was not an advanced one either she had plenty of time to think how she would decorate her table at her first "married" dinner party. In the first place the damask cloth was so white and shimmering that it seemed almost silvery, and suggested to her a white and silver decorative scheme. Her white candlesticks had white wax candies stuck in them, and she fashioned some gorgeous white and silver shades out of the silver paper usually used for wrapping cheap candies, and lace paper. For a centre piece she had a silvery mirror banked in green moss. On this stood a silver dish filled with asparagus vine, which had the advantage of being both effective and inexpensive. Long pieces of the same feathery green stuff trailed from the bowl over the white spread to the six places. At each place lay a name card of white, written in silver.

### THE DRESS.

The Fall Colors.—The most conspicuous shades among the new colors are blue and brown, though green will not lose its standing in the new goods. There are at least six or seven new shades of brown, all more or less of the yellowish order, the deepest of them being but little darker than chestnut. Of the blues, the very dark shades are falling somewhat into disfavor on account of the almost universal popularity of navy blue serge, and the shades most shown are of that indeterminate kind which suggests tinges of green or gray.

The Workingwoman's Dress.—The simplest dresses are now not only the most serviceable and the most economical, but are the prettiest and most fashionable. A plain skirt, made upon a gown form, a plain easy-fitting waist, with some simple trimmings on the sleeves, is the most suitable for a working dress, and is very inexpensive. An elaborately trimmed dress for such a purpose is not only a detriment to health, but shows wretched taste on the part of the wearer. Corsets can be let out by those who cannot afford to purchase proper corset-waists, and shoulder straps of strong muslin, doubled, can be easily added. To live under the tyranny of tight, ill-fitting and heavy clothes is as degrading to true womanhood as it is destructive to health.

Extra Waists.—The complete wardrobe always has two or three extra waists among its possessions. They are economical and lend variety to the other gowns. It is made of striped silk violet, pink and black, and fits the figure tightly to the waist line. There a full little basque is added, which is laid in plaits at the side. Wide revers in pink silk edged with a feather stitching in black fold back to display a black chiffon chemisette. A fluted collar and high gauntlet cuffs are also made of the pink silk. The special charm of this bodice is that it can be worn with so many different skirts. With a pink crepon skirt it is in perfect harmony. It looks well with a violet skirt trimmed with black lace, and think of its brightening power when combined with a skirt of black silk!

Fall Hats and Bonnets.—What odd shapes we find in the new hats and bonnets! Some of them are simply indescribable, and one needs an artist's pencil. There is a shovel shaped hat of satin straw bound with fine plaid velvet. The square front is turned backward, and in the center is a bunch of blue velvet cornflowers and a straggling bunch of wide velvet leaves. The crown and sides of the hat are literally covered with the flowers.

There are other queer shapes, one of them being scalloped into points, and above the pointed brim there are Mercury wings held by a bow of velvet. These wings are sometimes parrots', again doves', and then again it would be impossible to imagine what bird had worn them, as they will be made iridescent with peacocks' feathers such as grow on the neck, and humming birds' plumes. Some felt hats in pearl gray in the various harlequin shapes will be covered with white mistletoe or holly berries and leaves, so natural that it seems they must have grown there. Some of the Napoleonic and Revolutionary hats of felt, straw or velvet are very becoming and really artistic. One that pleased me was of modore satin straw turned up in a tricorne fashion. Tiny ostrich feathers curled over the brim instead of the rosettes so often seen, and two rich tips stood up in front. In the back there was a close brown velvet bow held by a square rhinestone buckle.

The wide ruffles for the neck, made of silk or velvet, are more elaborate than ever. Some are cut in a half circle and let to fall in natural folds. This is called the ripple, and it is very popular.

### THE KITCHEN.

Hickory-nut Drop Cake.—Hickory-nut drop cake is delicious when served fresh for luncheon. Take the yolks of two eggs and the whites of four, two cups of powdered sugar, one cup of sifted flour, two cups of rolled hickory-nuts and a little salt. Drop with a teaspoon in buttered tins a distance apart; bake in a moderate oven to a light brown. Care must be taken not to bake them too much.

How Old Are the Eggs.—To determine the exact age of eggs, dissolve about four ounces of common salt in a quart of pure water and then immerse the egg. If it be only a day or so old it will sink to the bottom, but if it be three days old it will float in the liquid, if more than five it comes to the surface and rises above in proportion to its increased age.

Banana Blanc Mange.—Into a quart of boiling milk stir four tablespoonfuls of corn starch wet with a little milk, and a quarter of a cupful of sugar. When it thickens set aside to cool. When properly cold stir in a small teaspoonful of extract of vanilla and two or three thinly sliced bananas.

Pickled Watermelon Rind.—To seven pounds of rind, which has been cut in fancy shapes and soaked in strong salt and water for two days, allow three pounds of sugar and one quart of vinegar. Scald the rinds in ginger tea, drain and drop in syrup, flavor with mace, cloves and cinnamon. Boil until the rind is tender. Put in jars, boil the syrup down, pour over and seal.

Frozen Custard.—Take one quart of milk, one quart of cream, six eggs and three cups of sugar beaten up with the yolks. Also one pint of fresh peaches cut up small. Heat the quart of milk almost to boiling and add it gradually to the beaten yolks and sugar. Whip in the frothed whites, return to the custard kettle and stir until it is a thick soft custard. Let it get perfectly cold, beat in the cream and freeze. If you let it freeze itself stir in the fruit after the second beating; if you turn the freezer then the custard will be like congealed mush.

### IN THE SICK ROOM.

### PRACTICAL HINTS TO THE NURSE.

Sickness is a calamity from which no flesh is exempt. There are balms for the wounds of the rich, but the poor must bear their own infirmities. With love, exquisite neatness, patience, encouragement, quiet and a little hospital learning, wondrous cures can be wrought.

Chewing will stop the ordinary nose-bleeding, and the shock of dropping a cold key or a handful of pennies down the back will often give relief. In case of a severe attack syringe the nasal cavity with cold salt water. If this does not stop the flow of blood, throw the head back, raise the arm up as far as possible, and apply cold sponges to the bridge of the nose and the back of the neck.

The quickest way to treat a burn or scald is to cover it with carron oil and flour and bandage with linen. In case of prostration from either accident administer a mild stimulant.

When a delicate person is fatigued and has no appetite, sponging the body with bathing whiskey, diluted alcohol or milk, will nourish the system and produce rest or refreshing sleep.

A bug in the ear may be drowned out with a little warm water. Apply with a sponge or syringe, and after each injection incline the head with a jerk so as to dislodge the contents of the cavity.

Distressing vomiting may be relieved by applying to the stomach a hot shingle or woollen pad brought from the oven.

In cases of sleeplessness as well as sickness frequent changing of pillows will have a soothing influence. The case should fit the pillow and be kept smooth. A change of night-gowns is restful, and the refreshing sense of cool water or some spicy toilet water, like lavender or orange flowers, applied to the forehead, throat, hands and feet cannot be overestimated.

A patient should never be raised suddenly to receive food, drink or medicine. Aside from distressing the sufferer there is danger of heart trouble. Place the arm under the pillow and gently force the invalid into a position between lying and sitting. Medicine cups and porcelain spoons are now in general use and save considerable unnecessary torture.

A piece of new muslin dipped in a glass of cool water will remove sordes from the teeth and cool the lips of a patient. Sore lips should be annointed with a little diluted water.

To induce a little baby or a grieving child to put out his tongue, drop a little sweet oil on the upper lip.

If a child has sore eyes, wring a sponge out of warm water containing a pinch of salt and trickle a stream on the inflamed lids, letting the water run towards the nose. As there is the danger of contagion the drying-towel should not be used by others.

Only bright, happy, healthly subjects should be discussed in the hearing of a sick or ailing person.

